# Kimura's Disease—A Rare Cause of Postauricular Swelling: A Case Report from the Hilly Region

**DOI:** 10.1055/s-0041-1742179

**Published:** 2022-01-17

**Authors:** Trilok C. Guleria, Mahender Singh, Vishal Singh, Ramesh K. Azad, Narender K. Mohindroo

**Affiliations:** 1 Department of Otolaryngology—Head and Neck Surgery, Dr Radhakrishnan Govt. Medical College, Hamirpur, Himachal Pradesh, India; 2 Department of Otolaryngology—Head and Neck Surgery, Indira Gandhi Medical College, Shimla, Himachal Pradesh, India; 3 Department of Otolaryngology—Head and Neck Surgery, Dr. Rajendra Prasad Govt. Medical College, Tanda, Himachal Pradesh, India

**Keywords:** chronic, postauricular, eosinophilia, lymphadenopathy, IgE

## Abstract

Kimura's disease (KD) is a chronic inflammatory disorder of the lymph node which is very rare in the Indian population. A 34-year-old female presented with left postauricular region swelling for the past 3 years at an outpatient department. On histopathological examination, it was diagnosed as KD. It should be kept in mind when treating a patient with lymphadenopathy and eosinophilia or a high immunoglobulin E level. This unique case report highlights this impressive clinical entity.

Kimura's disease (KD) was first described by Kim and Szetu in 1937 as an eosinophilic hyperplastic lymphogranuloma. The disease was known as KD since it was described by Kimura et al in 1948 in a manuscript titled “On the Unusual Granulation Combined with Hyperplastic Changes of Lymphatic Tissue. 1 2 It is endemic in Japan and China but sporadic cases have been reported worldwide. 3

KD is rare in India, with approximately 200 reported cases worldwide since its histopathological diagnosis. 4 The disease is characterized by the triad of painless subcutaneous masses, peripheral eosinophilia, and increased immunoglobulin (Ig) E levels. Recent studies have shown that KD occasionally shows the clonal proliferation of T-cells. 5 The diagnosis of KD is often difficult, and biopsy/excision of involved mass is necessary. Early and timely diagnosis of KD can avoid the unnecessary and expensive invasive diagnostic procedure, as it may mimic a neoplastic condition.

## Case Report

**Fig. 1:**
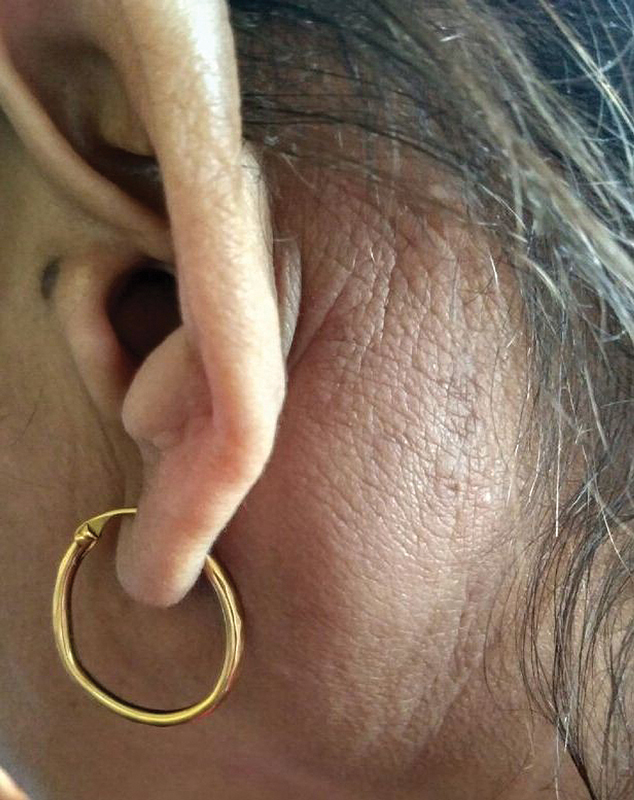
Preoperative photograph showing postauricular swelling left side.

A 34-year-old female presented to the ear, nose, and throat outpatient department for left-sided postauricular swelling for 3 years. There was no history of fever, any other swelling in the body, ear discharge, scalp infection, or weight loss. On local examination, swelling of size, 4 cm × 3 cm was present in the left postauricular area which was nontender, firm, nonfluctuant with normal skin overlying ( Fig. 1 ).

**Fig. 2:**
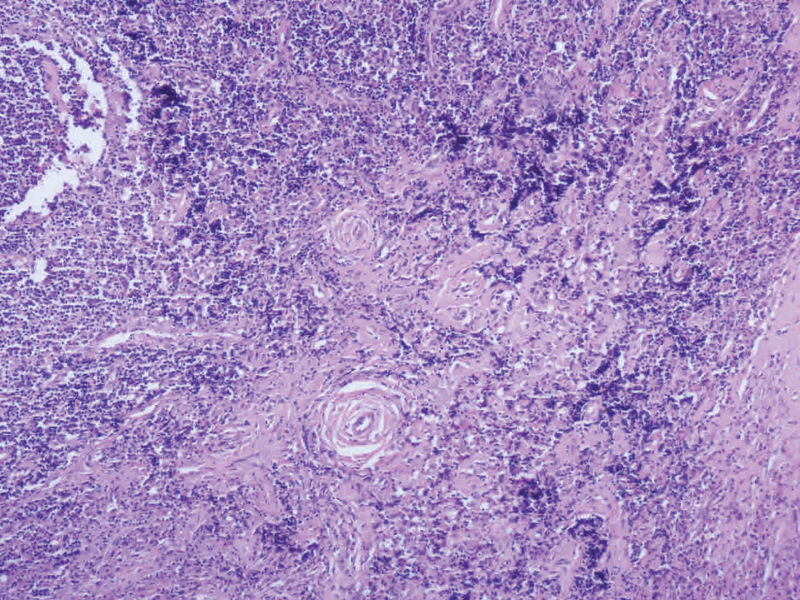
Histopathological picture of Kimura's disease (H&E ×10 magnification). Showing fibromuscular tissue with interspersed lobules of polymorphous population of lymphoid cells with fair numbers of eosinophils forming collection. Fair number of hyalinized blood vessels seen. Features were suggestive of Kimura's disease.

**Fig. 3:**
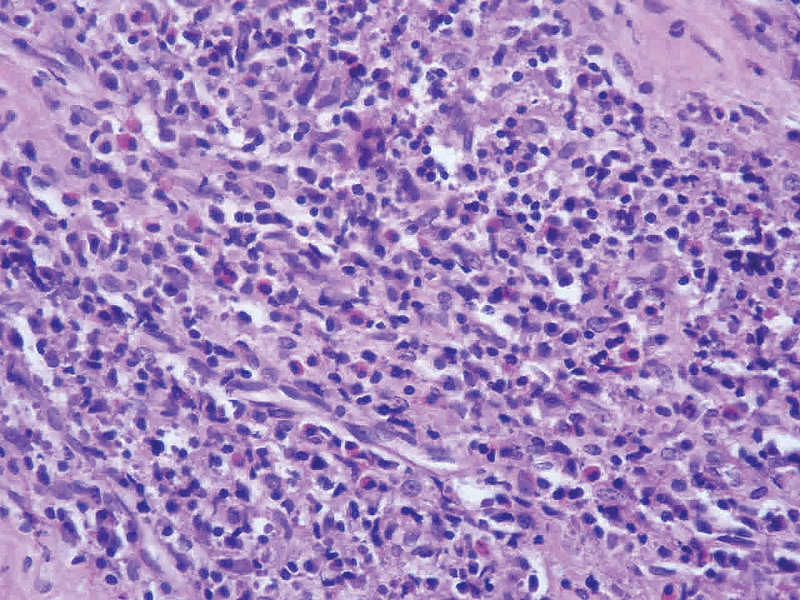
Histopathological picture of Kimura's disease (H&E ×40 magnification). Showing fibromuscular tissue with interspersed lobules of polymorphous population of lymphoid cells with fair numbers of eosinophils forming collection. Fair number of hyalinized blood vessels seen. Features were suggestive of Kimura's disease.

Peripheral blood smear showed 20% eosinophils. Renal function tests were within normal limits and there was no evidence of proteinuria. Chest radiograph was also within normal limits. Fine-needle aspiration cytology was suggestive of nonspecific reactive hyperplasia. Because of concern that the mass might be some lymphoproliferative disorder or neoplasm, an excision biopsy was taken. Histopathological examination of the mass showed fibromuscular tissue with interspersed lobules of the polymorphous population of lymphoid cells with fair numbers of eosinophils forming a collection. A fair number of hyalinized blood vessels were seen. Features were suggestive of KD ( Figs. 2 and 3 ). There was no recurrence in 1-year follow-up.

## Discussion

KD commonly presents with painless subcutaneous masses of insidious onset with lymphadenopathy in the head and neck region. It occasionally affects periauricular, axillary, epitrochlear, or inguinal nodes. In the second and third decades of life, the clinical triad of a subcutaneous nodule in the head and neck region, prominent peripheral eosinophilia, and highly elevated IgE is highly suggestive of KD, although it can become apparent at any age. 6

KD clinical course is commonly benign and self-limiting. It may be complicated by renal involvement. In 12 to 16% of cases, proteinuria may occur. The most common presentation is nephrotic syndrome. 7 A broad spectrum of histological lesions, such as minimal change disease or mesangioproliferative glomerulonephritis, membranous nephropathy, focal segmental glomerulosclerosis, IgA nephropathy, and IgM nephropathy, has been described to be associated with it. The lesions of KD usually precede the development of renal disease. Sometimes, it may present with renal involvement as initial presentation, before the appearance of subcutaneous lesions, leading to delay in diagnosis. 8 In our patient, renal functions were normal with no evidence of proteinuria.

The etiopathogenesis of KD is unclear, although it might be a self-limited autoimmune or allergic response triggered by an unknown stimulus. It has been postulated that a parasitic or viral trigger may alter T-cell immunoregulation or induce an IgE-mediated type-1 hypersensitivity resulting in the release of eosinophiliotrophic cytokines. 8 9 10

The differential diagnosis of KD is extensive and includes angiolymphoid hyperplasia with eosinophilia, Hodgkin's lymphoma, angioimmunoblastic T-cell lymphoma, Castleman's disease, florid follicular hyperplasia, Langerhans' cell histiocytosis, dermatopathic lymphadenopathy, lymphadenopathy of drug reactions, and parasitic lymphadenitis. 1

There is no consensus in the treatment of KD. Various treatment methods have been tried with variable results. Excision of the mass is the treatment of choice if the whole lesion can be removed, but local recurrence is common. Localized initial recurrence can be managed with repeated surgical excision. Systemic steroids may be indicated in cases complicated by nephrotic syndrome or frequent relapses. Radiation may be considered in cases refractory to surgical and medical therapy for recalcitrant and large tumors. 11

Cyclosporine, all-transretinoic acid with prednisolone, oral pentoxifylline, leflunomide, imatinib, vincristine, and azathioprine have been tried in the management for KD with variable results. 12 The choice of treatment method should be individualized. But unfortunately, recurrence is common almost with all the methods of treatment.

## Conclusion

KD is a rare chronic inflammatory disease commonly occurring in males. Although it is a rare disorder, it may be suspected in a patient with slowly progressive head and neck swelling or lymphadenopathy with eosinophilia or high IgE level. It is a distinctive clinicopathologic entity with variable responses to various treatment modalities. We present this impressive clinically entity in a young Indian female to add another case into the literature.
